# An observational study of dose dense chemotherapy with lipegfilgrastim support in early breast cancer

**DOI:** 10.1186/s12885-023-10603-0

**Published:** 2023-02-20

**Authors:** Ahmed Rashed, Orla M Fitzpatrick, David J Easty, Zac Coyne, Dearbhaile Collins, Victoria Mallet, Maciej Milewski, Keith Egan, Oscar S Breathnach, Liam Grogan, Bryan T Hennessy, Patrick G Morris

**Affiliations:** 1 Medical Oncology Department, Beaumont Hospital, Dublin, Ireland; 2 Cancer Clinical Trials and Research Unit, Beaumont RCSI Cancer Centre, Dublin, Ireland; 3 Medical Oncology Department, Our Lady of Lourdes Hospital, Drogheda, Co. Louth Ireland

**Keywords:** Breast cancer, lipegfilgrastim, Neutropenia, Doxorubicin, Cyclophosphamide

## Abstract

**Purpose:**

Breast cancer is one of the most prevalent malignant diseases in women. The development of dose dense chemotherapy regimens has improved clinical outcomes but has been associated with increased hematological toxicity. Currently there is a paucity of data on the use of lipegfilgrastim in dose dense AC treatment in early breast cancer. The purpose of this study was to assess the use of lipegfilgrastim in the treatment of early breast cancer and to examine the incidence of treatment-related neutropenia during the dose dense AC phase and subsequent paclitaxel treatment.

**Methods:**

This was a single arm, non-interventional, prospective study. The primary endpoint was to determine the rate of neutropenia defined as ANC of < 1.0 × 10^9^/L, during four cycles of dose dense AC with lipegfilgrastim support. The secondary endpoints were the incidence of febrile neutropenia, (temperature > 38 °C and ANC < 1.0 × 10^9^/L), treatment delays, premature treatment cessation and toxicity.

**Results:**

Forty-one participants were included in the study. Of the 160 planned dose dense AC treatments, 157 were administered, and 95% (152/160) of these were given on time. The rate of treatment delay was 5% (95% CI 2.2 to 9.9%) due to infection (4) and mucositis (1). Four (10%) patients developed febrile neutropenia. The most frequently occurring adverse event was grade 1 bone pain.

**Conclusion:**

Lipegfilgrastim is an effective option in the prophylaxis of chemotherapy-induced neutropenia, and its use in everyday anti-cancer treatment can be considered.

## Introduction

Breast cancer is one of the most prevalent malignant diseases in women, and improving its treatment and outcomes is of global public health concern [1]. Breast cancer rates vary between 19.7 and 152 cases per 100,000 women annually, totaling approximately 2 million cases worldwide [2]. The development of dose dense cytotoxic chemotherapy protocols, where treatment intervals are shortened has improved clinical outcomes, including all-cause mortality [3, 4]. These improved outcomes include fewer breast cancer recurrences, longer progression free survival, and reduced 10-year mortality. Doxorubicin and cyclophosphamide (AC) followed by paclitaxel (T) has been a commonly used regimen, particularly for patients with high-risk disease [5, 6]. The development of recombinant granulocyte colony-stimulating factor (G-CSF) facilitated the delivery of dose dense regimens [7].

The delivery of recombinant G-CSF in systemic anti-cancer therapy has evolved in parallel with changes in cytotoxic regimens [8]. Previously, short acting G-CSF, or filgrastim, was given as a daily subcutaneous dose for up to 7 days to allow neutrophil recovery. A significant advance was the development of Pegfilgrastim, a pegylated form of filgrastim, which is a longer acting G-CSF and require a single injection per cycle. It has shown improved efficacy in comparison to the non-pegylated formula, with lower incidences of febrile neutropenia, antibiotic use and hospital admissions and has formed the backbone of dose dense regimens in recent years [6, 9, 10]. Lipegfilgrastim is a pegylated long-acting covalent conjugate of filgrastim, which has previously been shown to be non-inferior to pegfilgrastim and also offers patients the convenience of a single injection [11, 12]. Older research with lipegfilgrastim in breast cancer has focused on doxorubicin/docetaxel chemotherapy.

Currently there is a paucity of data on the use of lipegfilgrastim in dose dense AC treatment in early breast cancer, which might be expected to be more myelosuppressive than doxorubicin/docetaxel. To prospectively assess the use of lipegfilgrastim in the treatment of early breast cancer a prospective study was conducted. A specific aim was to examine the incidence of treatment-related neutropenia during the dose dense AC phase and subsequent paclitaxel.

## Methods

This was a single arm, non-interventional, prospective study. This study was approved by the Institutional Review Board and registered with www.clinicaltrials.gov (Registration date 19/08/2015, registration number NCT02527317). Patients who were planned for dose dense AC every two weeks as adjuvant or neoadjuvant treatment were included. All patients were aged ≥ 18 years, had stage I-III breast cancer, ECOG performance status of 0–1 and adequate bone marrow function as defined by absolute neutrophil count (ANC) ≥ 1.0 × 10^9^/L and platelet count of ≥ 100 × 10^6^/L. Patients with HER2 positive disease, who were planned for the monoclonal antibody trastuzumab were included. Patients with prior exposure to chemotherapy, or G-CSF were excluded. This was chosen as a practical endpoint to reflect real world clinical practice, since treatment is usually delayed when the ANC is less than this threshold and the efficacy of dose dense regimens (compared to 3 weekly scheduling) relies on delivering chemotherapy two weekly. A sample size of 40 patients was chosen *a priori* as it was expected that 160 cycles (40 patients each receiving 4 cycles) would be likely to give a reasonable estimate of the efficacy of lipegfilgrastim in this setting. The grading of adverse events followed the Common Terminology Criteria for Adverse Events version 5.

The secondary endpoints were the incidence of febrile neutropenia, (temperature > 38 °C and ANC < 1.0 × 10^9^/L), treatment delays, premature treatment cessation and toxicity. Additional endpoints were to determine the rate of neutropenia, anemia and thrombocytopenia during weekly administration of paclitaxel given after dose dense AC.

All patients provided written informed consent and were treated with four cycles of doxorubicin (60 mg/m^2^) and cyclophosphamide (600 mg/m^2^) once every two weeks with lipegfilgrastim 6 mg subcutaneously on day 2. The numbers of patients treated in the adjuvant and neoadjuvant setting were not pre-specified as it was expected that the safety and efficacy are similar in both cohorts. Patients receiving paclitaxel were treated for 12 cycles once every week (80 mg/m^2^).

In accordance with Institutional guidelines, treatment was administered on schedule once the absolute neutrophil count was ≥ 1.0 × 10^9^/L and there was no other grade ≥ 3 toxicities. To allow for scheduling flexibility (e.g., around holidays) a window of +/- 2 working days was permitted when determining whether treatment was delivered “on time”. These treatments are considered part of standard clinical practice and no patient had their treatment changed because of this study. Data were collected from full blood counts performed for standard clinical indications prior to chemotherapy. Data were also collected on the incidence of febrile neutropenia, during dose dense AC chemotherapy (total duration of 2 months).

## Results

**Fig. 1:**
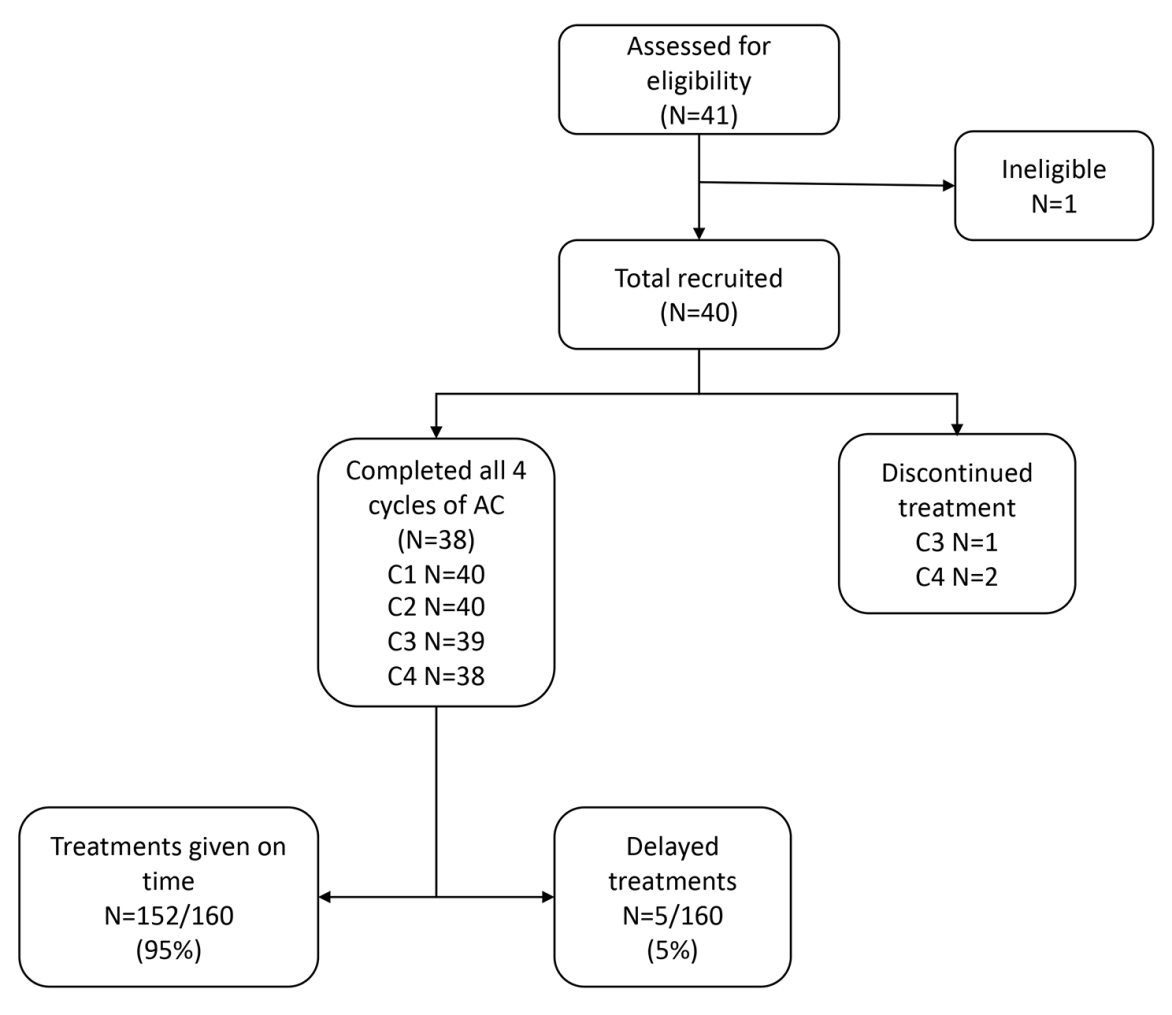
Patient eligibility, including number of treatments given per cycle with delays

Forty-one women with early-stage breast cancer were included in the study (Fig. 1) but one patient did not receive chemotherapy and was excluded. The first patient was consented on 21/10/2015. Hence there were 160 planned cycles of AC. One patient discontinued treatment after 2 cycles due to a prolonged lower respiratory tract infection, and one further patient did not receive the fourth cycle of treatment due to a prolonged episode febrile neutropenia, resulting in a total of 157 (98.1%, 157/160) treatments being administered. The majority (77%) of patients eligible had invasive ductal carcinoma and 10% had invasive lobular carcinoma and the median age was 52 years (Table 1.)

**Table 1:** Patient demographics

	N (%)
Age	
Median	52
Range	32–73
**Stage**	
II	19 (48%)
III	20 (50%)
Unknown	1 (2%)
**Histopathology**	
IDC	31 (77%)
ILC	4 (10%)
Other	5 (13%)
**Receptor Status**	
ER/PR positive, HER2 negative	25 (63%)
HER2 positive	9 (23%)
Triple negative	6 (14%)

IDC – Invasive Ductal Carcinoma. ILC – Invasive Lobular Carcinoma. ER – Estrogen Receptor. PR – progesterone receptor. HER2 – Human Epidermal growth factor receptor 2

**Fig. 2:**
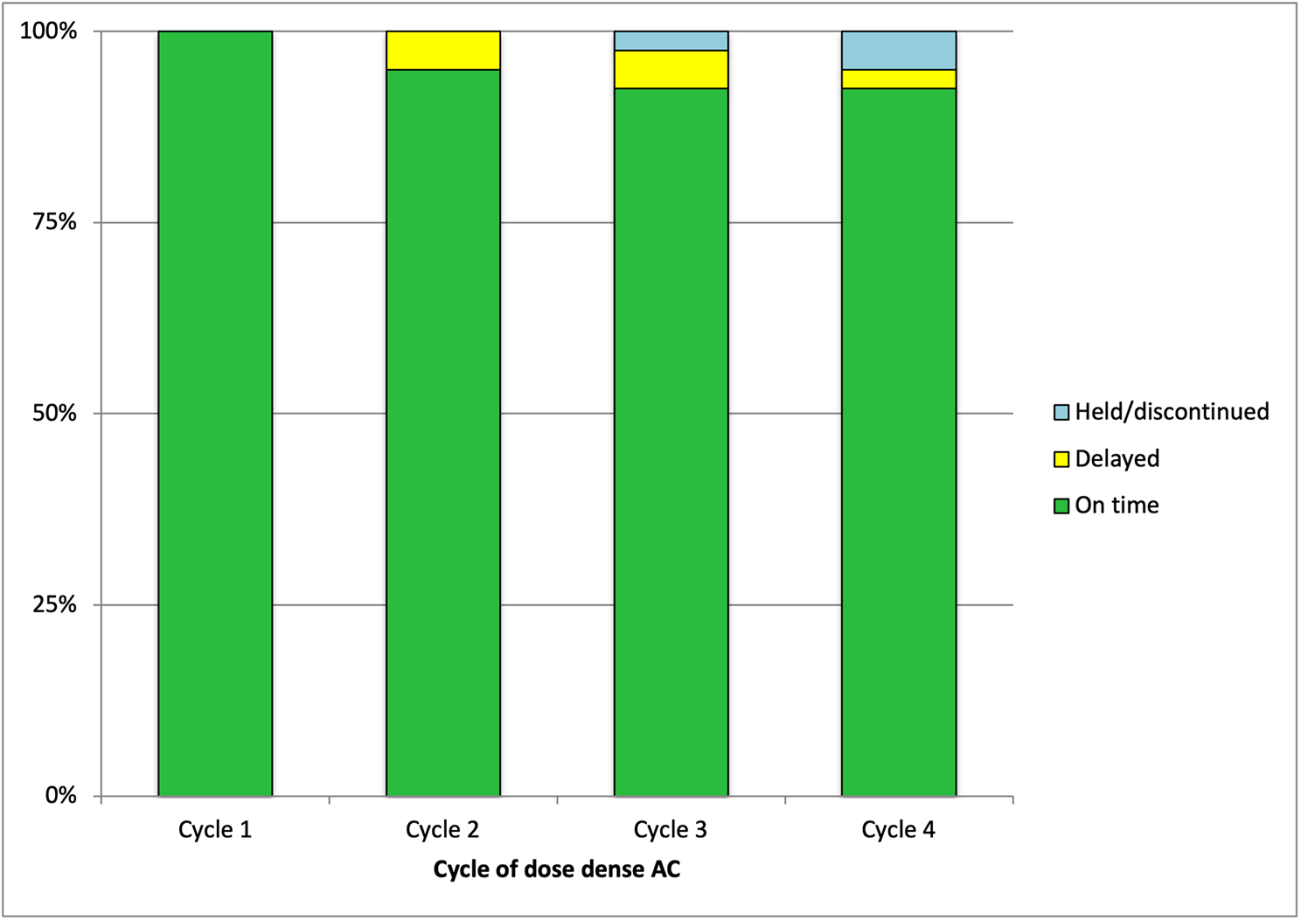
Proportion of dose dense AC delivered on time

Time delays in the administration of treatment were also assessed, by observing the difference between the treatment due date and the administration date (Fig. 2**)**. The vast majority (95%) of treatments were given on time (152/160). The rate of treatment delay was 5% (95% CI 2.2 to 9.9%). Of the 5 treatments that were administered outside this treatment window, 4 delays (2.5%) were infection related, and 1 (0.6%) was due to mucositis. Two (1.25%) of these delays occurred at cycle 2 of dose dense AC; one 7-day delay was due to a combined neutropenia and LRTI, and the second delay was due to hospital admission with an abscess requiring prolonged antibiotics, resulting in a 9-day delay of treatment. Two further delays (1.25%) occurred at cycle 3; an 8-day delay due to lower respiratory tract infection, and a 4-day delay due to a central line infection. One treatment (0.6%) was delayed at cycle 4 for 7 days due to severe mucositis.

**Fig. 3:**
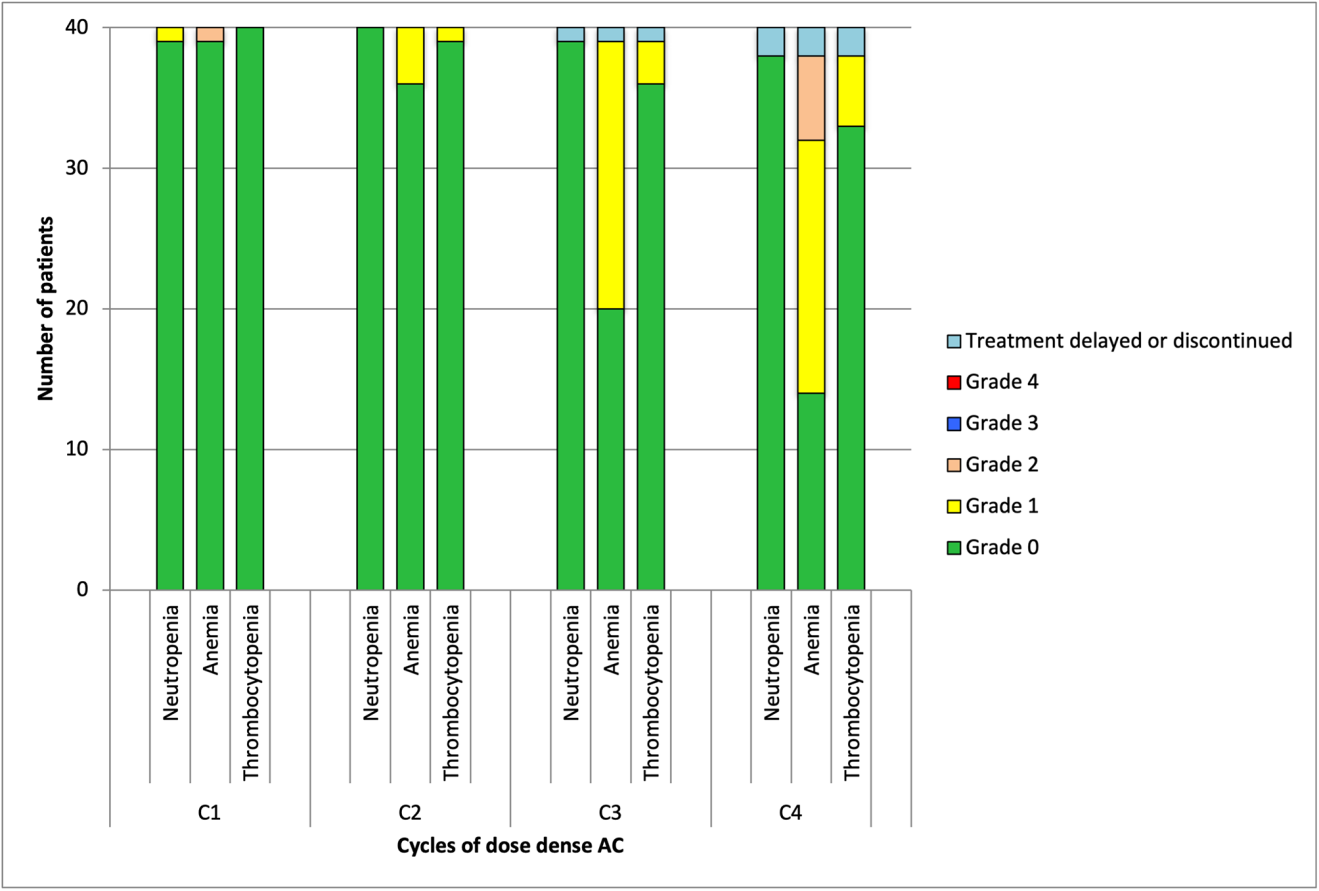
Hematological Toxicity by cycle of dose dense AC

The primary endpoint was to determine the rate of neutropenia defined as ANC of < 1.0 × 10^9^/L, following four cycles of dose dense AC with lipegfilgrastim support. The incidence of neutropenia, anemia and thrombocytopenia prior to each cycle of AC are shown in Fig. 3. Prior to treatment being administered, no grade 2, 3 or 4 neutropenia were identified. One patient (2.5%) met the criteria for grade 1 neutropenia at cycle 1. No grade 3 or 4 anemias, or grade 2, 3, or 4 thrombocytopenia were identified throughout the AC phase of the study.

During the duration of the study, 4 patients (10%) developed febrile neutropenia. Of these 4 episodes, 1 occurred post cycle 1, 2 episodes occurred post cycle 3, and 1 occurred post cycle 4 of treatment. These events were identified by additional sets of full blood counts, performed following clinical review of patients with temperatures > 38 C. The event post cycle 1 was defined as a grade 4 neutropenia which resolved in less than 1 week. The second patient post cycle 3 developed a grade 4 neutropenia, which took 2 weeks to recover, resulting in the final AC treatment being held. Lastly, one episode of febrile neutropenia occurred after cycle 4 of AC and was a grade 4 neutropenia which resolved within one week.

Other targeted adverse events which did not affect the administration of dose dense AC chemotherapy are recorded in Table 2. The most frequently occurring adverse event was grade 1 bone pain, defined as mild pain, but not limiting activities of daily living. There were no grade 3 or 4 non-hematological adverse events throughout this study.

**Table 2:** Adverse events of interest

Adverse Event	ddAC cycle number	Grade I N (%)	Grade II N (%)
Bone pain			
	1	0 (0%)	1 (2.5%)
	2	12 (30%)	2 (5%)
	3	10 (25%)	1 (2.5%)
	4	7 (17.5%)	2 (5%)
**Myalgia**			
	1	0 (0%)	0 (0%)
	2	3 (7.5%)	1 (2.5%)
	3	2 (5%)	0 (0%)
	4	3 (7.5%)	0 (0%)
**Injection site reaction**			
	1	0 (0%)	0 (0%)
	2	1 (2.5%)	0 (0%)
	3	0 (0%)	0 (0%)
	4	0 (0%)	0 (0%)

**Fig. 4:**
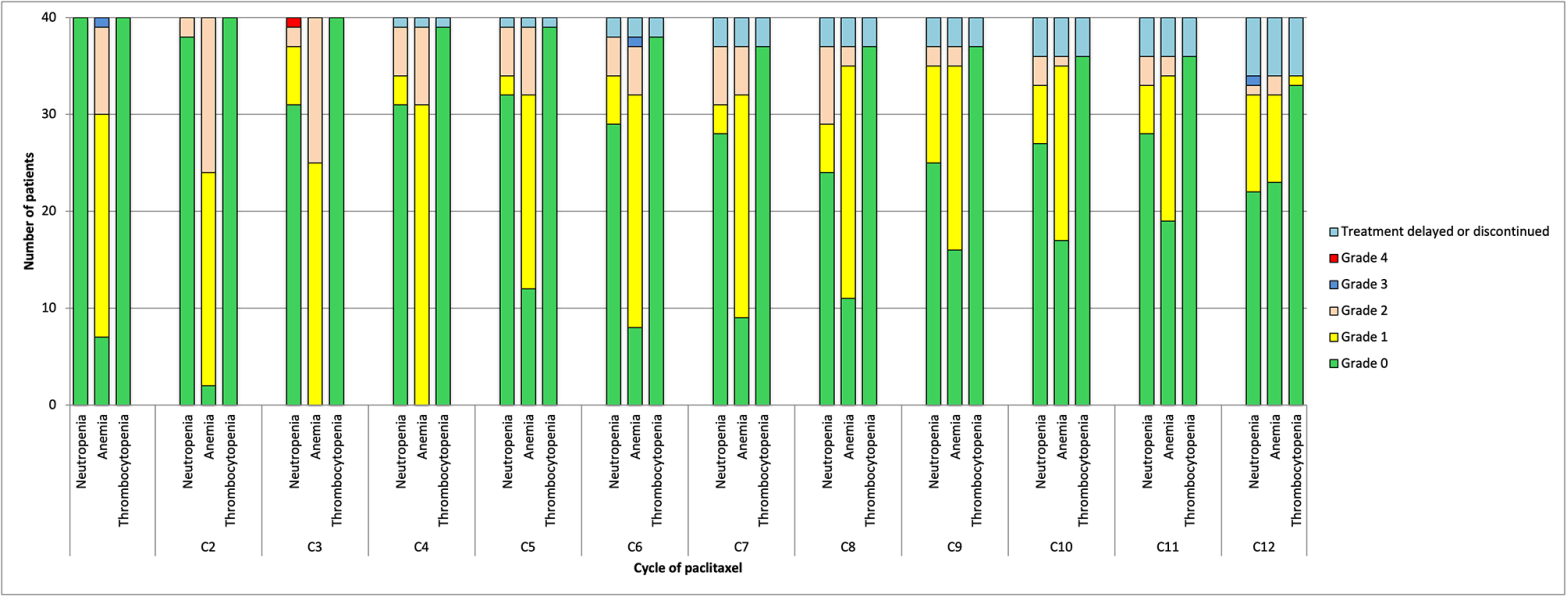
Hematological toxicity by cycle of paclitaxel

During the subsequent phase, all 40 patients were planned for 12 cycles of paclitaxel, totaling 480 treatments. Of the planned treatments, 94.4% were administered (453/480). A total of 27 treatments (5.6%) were held or discontinued due to adverse events including pneumonitis, peripheral neuropathy, inpatient admission for breast implant infection, and a prolonged infection. The hematological adverse events during this phase are summarized in Fig. 4. There was 1 episode (0.2%) of grade 4 neutropenia at cycle 3, and 1 episode of grade 3 neutropenia at cycle 12. During the 12 cycles, there was 1 episode of febrile neutropenia. There was no grade 2, 3 or 4 thrombocytopenia. There were 2 episodes (0.4%) of grade 3 anemia, but no grade 4 anemia during this treatment phase.

## Discussion

This study has successfully shown that the administration of lipegfilgrastim allows dose dense AC chemotherapy to be delivered on time. Of the 160 treatments scheduled as part of this study, only 3 (1.8%) treatments were held and 95% (152/160) planned treatments given were administered on time. In addition, patients who did not experience neutropenia during cycle 1, were unlikely to subsequently develop neutropenia (Figs. 1 and 3). In comparison to previous studies, we have demonstrated that lipegfilgrastim prophylaxis is associated with low rates of neutropenia during the dose dense phase of treatment. This study shows a low incidence of adverse effects, reinforcing the suitability of using lipegfilgrastim in real world settings.

Although a 10% incidence rate of febrile neutropenia is documented here, lipegfilgrastim has been previously shown to have rates as low as 2.1–4.5% in other studies assessing breast cancer [13]. As a smaller sample of women with early breast cancer were eligible for inclusion from the medical oncology department in our center, we do take into account the limitations generalizing the results from a smaller sample size [14, 15]. The fact that 95% of planned doses of dose dense AC were delivered on schedule does offer reassurance that lipegfilgrastim is an effective treatment option.

The rate of neutropenia (< 1.0 × 10^9^/L), during four cycles of dose dense AC was chosen as the primary endpoint as a practical surrogate of toxicity. Importantly, in every day clinical practice treatment is usually delayed when the ANC is less than this threshold. The efficacy of dose dense regimens (compared to 3-weekly scheduling) relied on the delivery of chemotherapy two-weekly. Hence this endpoint was chosen a priori when the study was designed. However, there are clear limitations with this study design. Since neutrophil counts were not assessed daily, it is unknown whether many patients developed neutropenia between chemotherapy doses and the duration of any resulting neutropenia that occurred. Although, this may not have made any practical difference to the patients in this study, as many did not develop febrile neutropenia, it is possible that greater toxicity would have been seen if a larger sample size had been enrolled with more frequent blood test monitoring.

The use of lipegfilgrastim is increasing, both in breast cancer and the treatment of other malignancies. It has repeatedly shown a minimal side effect profile and has been associated with low incidences of febrile neutropenia [16]. Bone pain and myalgia are the most documented adverse events that occur, irrespective of cancer type, including breast cancer, prostate cancer, lung cancer and non-Hodgkin’s lymphoma [16]. The positive effects of lipegfilgrastim have been demonstrated in patients with lung cancer, again reinforcing that its use as a prophylactic agent is associated with reduced rates of severe neutropenia and reduced time to absolute neutrophil count recovery [17].

This research shows that lipegfilgrastim is an effective option in the prophylaxis of chemotherapy-induced neutropenia, and its use in everyday anti-cancer treatment can be considered as part of dose dense AC regimens.

## Data Availability

Datasets used and/or analysed during this study are available from the corresponding author on reasonable request.

## Declarations

### Trial Registration Number

NCT02527317.

## References

[1] Toriola AT, Colditz GA. Trends in breast cancer incidence and mortality in the United States: implications for prevention [Internet]. Vol. 138, Breast Cancer Research and Treatment. 2013. p. 665–73. Available from: 10.1007/s10549-013-2500-7 10.1007/s10549-013-2500-7 23546552

[2] Heer E, Harper A, Escandor N, Sung H, McCormack V, Fidler-Benaoudia MM. Global burden and trends in premenopausal and postmenopausal breast cancer: a population-based study [Internet]. Vol. 8, The Lancet Global Health. 2020. p. e1027–37. Available from: 10.1016/s2214-109x(20)30215-1 10.1016/S2214-109X(20)30215-1 32710860

[3] Early Breast Cancer Trialists’ Collaborative Group (EBCTCG). Increasing the dose intensity of chemotherapy by more frequent administration or sequential scheduling: a patient-level meta-analysis of 37 298 women with early breast cancer in 26 randomised trials. Lancet. 2019 Apr;6(10179):1440–52. 10.1016/S0140-6736(18)33137-4 PMC6451189 30739743

[4] Citron ML, Berry DA, Cirrincione C, Hudis C, Winer EP, Gradishar WJ et al. Randomized trial of dose-dense versus conventionally scheduled and sequential versus concurrent combination chemotherapy as postoperative adjuvant treatment of node-positive primary breast cancer: first report of Intergroup Trial C9741/Cancer and Leukemia Group B Trial 9741. J Clin Oncol. 2003 Apr 15;21(8):1431–9. 10.1200/JCO.2003.09.081 12668651

[5] Fujii T, Le Du F, Xiao L, Kogawa T, Barcenas CH, Alvarez RH, et al. Effectiveness of an adjuvant chemotherapy regimen for early-stage breast Cancer: a systematic review and network Meta-analysis. JAMA Oncol. 2015 Dec;1(9):1311–8. 10.1001/jamaoncol.2015.3062 PMC5575939 26402167

[6] Morris PG, Dickler M, McArthur HL, Traina T, Sugarman S, Lin N, et al. Dose-dense adjuvant doxorubicin and cyclophosphamide is not associated with frequent short-term changes in left ventricular ejection fraction. J Clin Oncol. 2009 Dec;20(36):6117–23. 10.1200/JCO.2008.20.2952 PMC3664032 19901120

[7] Aapro M, Crawford J, Kamioner D. Prophylaxis of chemotherapy-induced febrile neutropenia with granulocyte colony-stimulating factors: where are we now? Support Care Cancer. 2010 May;18(5):529–41. 10.1007/s00520-010-0816-y PMC2846279 20191292

[8] Veitch Z, Khan OF, Tilley D, Tang PA, Ribnikar D, Stewart DA et al. Impact of Cumulative Chemotherapy Dose on Survival With Adjuvant FEC-D Chemotherapy for Breast Cancer. J Natl Compr Canc Netw. 2019 Aug 1;17(8):957–67. 10.6004/jnccn.2019.7286 31390594

[9] Pfeil AM, Allcott K, Pettengell R, von Minckwitz G, Schwenkglenks M, Szabo Z. Efficacy, effectiveness and safety of long-acting granulocyte colony-stimulating factors for prophylaxis of chemotherapy-induced neutropenia in patients with cancer: a systematic review. Support Care Cancer. 2015 Feb;23(2):525–45. 10.1007/s00520-014-2457-z 25284721

[10] Morris PG, Hudis CA. Trastuzumab-related cardiotoxicity following anthracycline-based adjuvant chemotherapy: how worried should we be?J Clin Oncol. 2010 Jul20;28(21):3407–10. 10.1200/JCO.2009.26.0125 20530269

[11] Bondarenko I, Gladkov OA, Elsaesser R, Buchner A, Bias P. Efficacy and safety of lipegfilgrastim versus pegfilgrastim: a randomized, multicenter, active-control phase 3 trial in patients with breast cancer receiving doxorubicin/docetaxel chemotherapy. BMC Cancer. 2013 Aug;14:13:386. 10.1186/1471-2407-13-386 PMC3751756 23945072

[12] Hoggatt J, Tate TA, Pelus LM. Role of lipegfilgrastim in the management of chemotherapy-induced neutropenia. Int J Nanomedicine. 2015 Apr;1:10:2647–52. 10.2147/IJN.S55796 PMC4388090 25878498

[13] Kiechle M, Schem C, Lüftner D, Hamann X, Jünemann R, Tölg M et al. Prophylaxis with lipegfilgrastim in patients with primary breast cancer receiving dose dense chemotherapy: Results from the German NIS NADENS [Internet]. Vol. 30, Annals of Oncology. 2019. p. v738. Available from: 10.1093/annonc/mdz265.061

[14] Faber J, Fonseca LM. How sample size influences research outcomes [Internet]. Vol. 19, Dental Press Journal of Orthodontics. 2014. p. 27–9. Available from: 10.1590/2176-9451.19.4.027-029.ebo 10.1590/2176-9451.19.4.027-029.ebo PMC4296634 25279518

[15] Kang M, Ragan BG, Park J-H. Issues in outcomes research: an overview of randomization techniques for clinical trials.J Athl Train. 2008Apr;43(2):215–21. 10.4085/1062-6050-43.2.215 PMC2267325 18345348

[16] Steger G, Pichler P, Airoldi M, Mazza P, Fontaine C, Timmer Bonte J et al. Use of lipegfilgrastim for the prophylaxis of chemotherapy-induced neutropenia: Pan-European non-interventional study [Internet]. Vol. 29, Annals of Oncology. 2018. p. viii607–8. Available from: 10.1093/annonc/mdy300.014

[17] Guariglia R, Martorelli MC, Lerose R, Telesca D, Milella MR, Musto P. Lipegfilgrastim in the management of chemotherapy-induced neutropenia of cancer patients.Biologics. 2016 Jan22;10:1–8. 10.2147/BTT.S58597 PMC4730998 26858523

